# A Novel Maximum Entropy Markov Model for Human Facial Expression Recognition

**DOI:** 10.1371/journal.pone.0162702

**Published:** 2016-09-16

**Authors:** Muhammad Hameed Siddiqi, Md. Golam Rabiul Alam, Choong Seon Hong, Adil Mehmood Khan, Hyunseung Choo

**Affiliations:** 1 College of Information and Communication Engineering, Sungkyunkwan University, Suwon-si, Gyeonggi-do, Rep. of Korea; 2 Department of Computer Engineering, Kyung Hee University, Suwon, Rep. of Korea; 3 Department of Computer Science, Innopolis University, Kazan, Russia; Jiangnan University, CHINA

## Abstract

Research in video based FER systems has exploded in the past decade. However, most of the previous methods work well when they are trained and tested on the same dataset. Illumination settings, image resolution, camera angle, and physical characteristics of the people differ from one dataset to another. Considering a single dataset keeps the variance, which results from differences, to a minimum. Having a robust FER system, which can work across several datasets, is thus highly desirable. The aim of this work is to design, implement, and validate such a system using different datasets. In this regard, the major contribution is made at the recognition module which uses the maximum entropy Markov model (MEMM) for expression recognition. In this model, the states of the human expressions are modeled as the states of an MEMM, by considering the video-sensor observations as the observations of MEMM. A modified Viterbi is utilized to generate the most probable expression state sequence based on such observations. Lastly, an algorithm is designed which predicts the expression state from the generated state sequence. Performance is compared against several existing state-of-the-art FER systems on six publicly available datasets. A weighted average accuracy of 97% is achieved across all datasets.

## Introduction

Knowledge about each other’s emotional states is important for effective communication among humans. They are responsive to each other’s emotions, and computers should gain this ability, too. Several scientific studies have been carried out to automatically detect human emotions in various fields. These include human-computer interaction [1, 2], psychology and cognitive sciences [3], access control and surveillance systems [4], and driver state surveillance. Physiological state of human body, such as blood pressure, heart rate, speech etc., is one way of monitoring someone’s emotions. Emotion recognition by recognizing facial expression offers a simple yet effective alternative [5–8].

**Fig 1:**
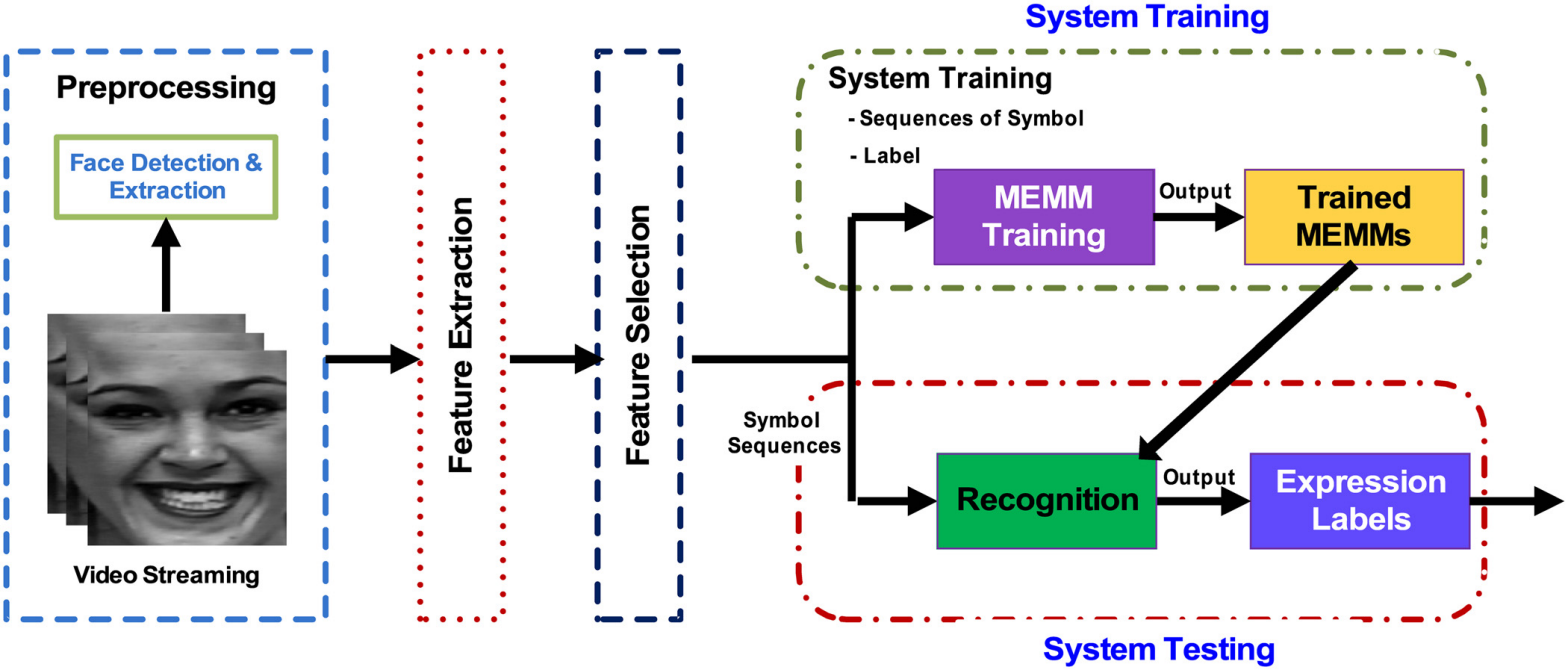
General flow diagram for a typical facial expression recognition (FER) system.

A typical facial expression recognition (FER) system performs four tasks. These include: preprocessing of video data, feature extraction, feature selection, and recognition, as shown in Fig 1. The preprocessing module processes the video frames to remove noise, detects facial boundaries, and performs face segmentation. The segmented facial region is processed by the feature extraction module to extract distinguishing features for each type of expression, which are then quantified as discrete symbols [9]. The feature selection module selects a subset of extracted features using techniques such as linear discriminant analysis. Finally, the recognizer module uses a trained classifier on the selected features to recognize the expression in the incoming video stream.

Previous studies in FER have mostly focused on the use of traditional learning methods in the recognizer module [10]. These include artificial neural networks (ANN), Gaussian mixture model (GMM), support vector machine (SVM), hidden Morkov model (HMM), deep learning methods, and hidden conditional random fields. Among these, HMM is the most commonly used learner for FER problems. However, as stated by [7, 11–13], the main weakness with HMM is its assumption that the current state depends on only the previous state.

Having these limitations and lack of improvement in HMM learning model, this paper investigates the use maximum entropy Markov model (MEMM) for FER. More specifically, in the proposed method the video observations are considered to be the observations of MEMM, and the facial expressions are modeled as the states of MEMM. A modified Viterbi is then used to generate the most probable expression state sequence based on modeled observations. Finally, the expression state is predicted from the most likely state sequence. It is also investigated and shown that the existing models are limited due to their independent assumptions which may result in decreasing the classification accuracy. For feature extraction and selection wavelet transform coupled with optical flow and stepwise linear discriminant analysis (SWLDA) are used, respectively. The proposed approach is tested and validated on six publicly available datasets. The average recognition accuracy is 97% across all the datasets. To the best of our knowledge, it is the first time that MEMM model is being utilized as a classifier for FER systems.

## Related Works

This section summarizes different classification methods that have been used in existing studies. For instance, artificial neural networks (ANNs) were used by [14, 15] in their work on FER. The major problem with ANNs is their high computational complexity. They may suffer from the problem of local minima as well [7].

Other systems, including [16–19] achieved good recognition performance by utilizing support vector machines (SVMs). However, SVM does not exploit temporal dependencies between adjacent video frames and each frame is processed statistically independent of others [7]. Similarly, Gaussian mixture model (GMM) was employed by [20–22] in their respective systems. However, GMM lacks ability to model abrupt changes, which limits its applicability for recognizing spontaneous expressions [23].

Different kinds of facial expressions were recognized by [24, 25] using decision trees. The memory requirements of a decision tree-based classifier are usually high. In addition to this, the patterns in a decision tree are defined on expectations and these expectations could be illogical, which could result in error-prone decision trees. Although, a decision tree follows a pattern matching for events and relationships between them, it may not be possible to cover all the combinations. Such oversights can lead to bad decisions, which shows the limitation of decision trees. [26].

Some works, such as [27, 28] have employed bayesian networks-based classifiers. However, a bayesian network-based classifier requires prior knowledge. Having limited or incorrect prior knowledge degrades the recognition performance. Moreover, it is very difficult for bayesian networks to handle continuous data [29].

As stated in [7, 30], the most commonly used learning method for FER is the HMM. It offers advantage of handling sequential data when frame-level features are used. In such a case, vector-based classifiers, e.g., GMM, ANN, SVM, decision tree, and bayes classifier, do not perform well. However, HMM has a well-known problem: it assumes that the current state depends only on the previous state, due to which these two states must occur consecutively in the observation sequence. This assumption does not hold in reality. To solve this, non-generative models such as conditional random fields (CRF) [31] and hidden conditional random fields (HCRF) [7, 11, 13] were proposed. HCRF is an extension of CRF to learn hidden structure of sequential data through hidden states. Both of them use global normalization instead of per-state normalization. This allows for weighted scores and makes the parameter space larger than that of HMM. However, HCRF requires explicitly involving the full covariance Gaussian distribution in the observation level which may cause the complexity issue [7].

## Materials and Methods

The details of each component of the proposed FER system is as follows.

### Preprocessing

Global histogram equalization (GHE) [5] is used to improve the image quality. GHE does that by increasing the dynamic range of the intensity using the histogram of the whole image. It obtains the scale factor from the normalized cumulative distribution of the brightness distribution of the original image and multiplies this scale factor by the original image to redistribute the intensity [32]. GHE finds the running sum of the histogram values and then normalizes it by dividing it by the total number of pixels. This value is then multiplied by the maximum gray-level value and then mapped onto the previous values in a one-to-one correspondence [32].

For the face detection and extraction, active contour (AC) based model is used [30]. This method automatically detects and extracts human faces from the expression frames, which is based on level sets integrated with two energy functions: Chan-Vese (CV) energy function to remove the dissimilarities within a face, and Bhattacharyya distance function to maximize the distance between the face and background.

### Feature Extraction and Selection

In order to represent movable parts of the face, features are extracted by applying the wavelet transform on the extracted facial regions. More specifically, the symlet wavelet transform coupled with optical flow is used. The former helps in diminishing the noise, whereas the latter extracts the facial movement features.

In order to remove any redundancy in the feature space,a non-linear feature selection method called stepwise linear discriminant analysis (SWLDA) is applied to the selected feature space. SWLDA selects the most informative features a forward selection model and removes the irrelevant features through a backward regression model. Further details are available in [30].

### Proposed Model

#### Details of the Maximum Entropy Markov Model (MEMM)

**Fig 2:**
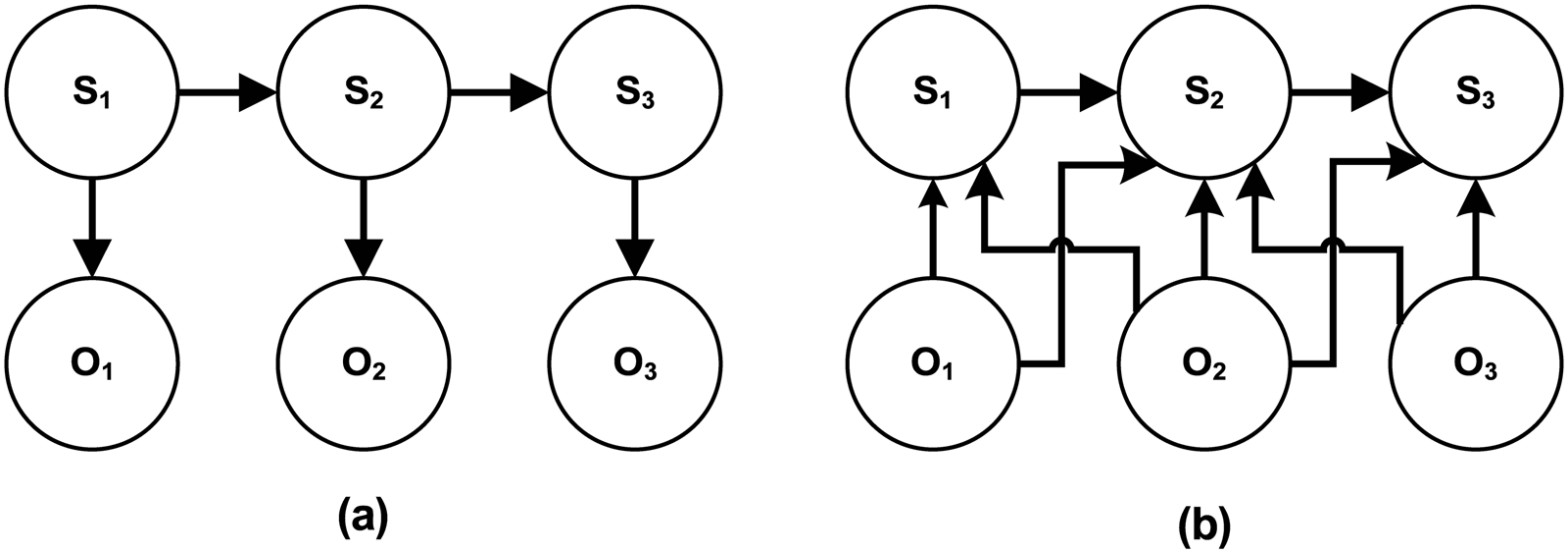
(a) shows the dependency graph of HMM, while (b) presents the dependency graph of MEMM.

As mentioned earlier, in this work the expression states are modeled as MEMM, as it is one of the best candidates for modeling the sequential states and observations similar to HMM. In generative HMM, the joint probability is used to determine the maximum likelihood of observation sequence. On the other hand, in discriminative MEMM, conditional probability is used to predict the state sequence from the observation sequence [33]. The dependency among the states and observations in HMM and MEMM are presented by the dependency graph shown in Fig 2.

**Fig 3:**
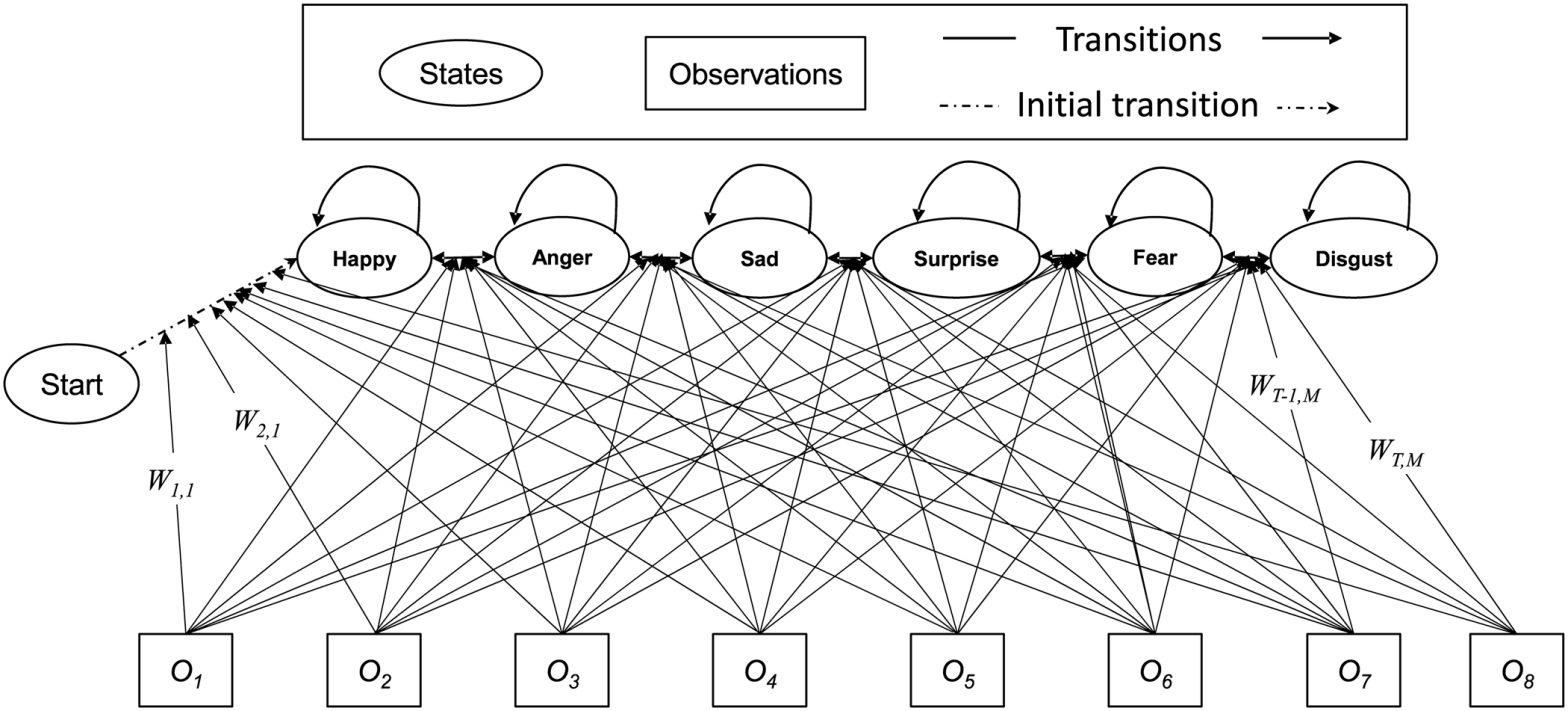
MEMM based on expression state model for FER system.

Fig 3 presents the *M* state MEMM model. The set of states is defined as the facial expressions Ψ = {*χ*_1_, *χ*_2_, …, *χ*_*M*_} = {Happy, Anger, Sad, Surprise, Fear, Disgust}. The corresponding frame observations are represented by the set Φ = {*φ*_1_, *φ*_2_, …, *φ*_ℑ_}, where ℑ observation ranking in time. Each *φ*_*i*_ is the vector of observed discriminative features {*δ*_1_, *δ*_2_, …, *δ*_*n*_}, which are extracted from the expression frames at time slot *t*_*i*_. Finally, ℵ is the total number discriminative features. Now the primal objective is to determine the most likely state sequence *L* = {*l*_1_, *l*_2_, …, *l*_*p*_} ∈ Ψ based on the current sequential observations Φ for the duration ℑ.

To generate the most likely state sequence, HMM requires transition probability *P* (Ψ_*i*_|Ψ_*i*−1_), emission probability *P* (Φ_*i*_|Ψ_*i*_), and initial probability *P* (Ψ_*i*_). On the other hand, MEMM requires a single function *P* (Ψ_*i*_|Ψ_*i*−1_, Φ_*i*_), which is easily obtainable from the maximum entropy model, as discussed in next section. These properties of MEMM is the reason that this work uses it to model expression states for determining the hidden expression state sequences.

#### Learning and Parameter Estimation in MEMM

Various methods exist in literature for estimating the parameters of MEMM, which are thoroughly described in [33]. This work utilizes the maximum entropy (MaxEnt: Ω) model (1) to estimate the transition probability from state Ψ_*i*−1_ to Ψ_*i*_ based on the observation Φ.

$$P\left(\Psi_{i}|\Psi_{i-1},\Phi_{i}\right)=e^{\sum_{k=1}^{\chi }\zeta_{k}\delta_{k}}$$ (1)

where *δ*_*k*_ is the feature value of observations of the training dataset considering *χ* features in total, *ζ*_*k*_ is the trainable weights of the multinomial logistic regression.

Now to fulfill the probability axiom of summation of probabilities of whole state space should be equal to 1. Therefore, the right hand side of Eq(1) is is normalized through a normalization factor ℜ to make the left hand side as a probability distribution of Ψ.

$$P\left(\Psi_{i}|\Psi_{i-1},\Phi_{i}\right)=\frac{e^{\sum_{k=1}^{\chi }\zeta_{k}\delta_{k}}}{ℜ}$$ (2)

$$P\left(\Psi_{i}|\Psi_{i-1},\Phi_{i}\right)=\frac{e^{\sum_{k=1}^{\chi }\zeta_{k}\delta_{k}}}{\sum_{\Psi }P\left(\Psi_{i}|\Psi_{i-1},\Phi_{i}\right)}$$ (3)

$$P\left(\Psi_{i}|\Psi_{i-1},\Phi_{i}\right)=\frac{e^{\sum_{k=1}^{\chi }\zeta_{k}\delta_{k}}}{\sum_{\Psi }e^{\sum_{k=1}^{\chi -1}\zeta_{k}\delta_{k}}}$$ (4)

According to Eq(4), to find out *P* (Ψ_*i*_|Ψ_*i*−1_, Φ_*i*_) the (MaxEnt: Ω) parameter *ζ*_*k*_ is now the major concern as the feature parameter *δ*_*k*_ is already known from the training dataset. Based on the MEMM modeling the facial expression classes are considered as the states of MEMM. To define the facial expression class level, the probability of the defined class should be greater than other facial expression classes. Therefore, maximization of *P* (Ψ_*i*_|Ψ_*i*−1_, Φ_*i*_) through parameter *ζ* is formulated as the following optimization problem Eq (5).

$$\hat{\zeta }=\underset{\zeta }{argmax}\frac{e^{\sum_{k=1}^{\chi }\zeta_{k}\delta_{k}}}{\sum_{\Psi }e^{\sum_{k=1}^{\chi -1}\zeta_{k}\delta_{k}}}$$ (5)

By assuming total *D* instances in training dataset and considering log likelihood probability, Eq (5) can be written as in Eq (6).

$$\hat{\zeta }=\underset{\zeta }{argmax}\sum_{j}^{D}log\frac{e^{\sum_{k=1}^{\chi }\zeta_{k}\delta_{k}^{\left(j\right)}}}{\sum_{\Psi }e^{\sum_{k=1}^{\chi -1}\zeta_{k}\delta_{k}^{\left(j\right)}}}$$ (6)

Afterwards, the regularization is used to penalize the large values of parameter *ζ*.

$$\hat{\zeta }=\underset{\zeta }{argmax}\sum_{j}^{D}log\frac{e^{\sum_{k=1}^{\chi }\zeta_{k}\delta_{k}^{\left(j\right)}}}{\sum_{\Psi }e^{\sum_{k=1}^{\chi -1}\zeta_{k}\delta_{k}^{\left(j\right)}}}-\beta *R\left(\zeta \right)$$ (7)

Here, the Gaussian distribution *N*(*μ*, *σ*^2^) of parameter *ζ* is used for regularization as shown in Eq (8).

$$\hat{\zeta }=\underset{\zeta }{argmax}\sum_{j}^{D}log\frac{e^{\sum_{k=1}^{\chi }\zeta_{k}\delta_{k}^{\left(j\right)}}}{\sum_{\Psi }e^{\sum_{k=1}^{\chi -1}\zeta_{k}\delta_{k}^{\left(j\right)}}}-\sum_{k}^{\chi }\frac{{\left(\zeta_{k}-\mu_{k}\right)}^{2}}{2\sigma_{k}^{2}}$$ (8)

As Eq (8) is a *log* − *sum* exponential equation, the popular Broyden Fletcher Goldfarb Shanno (BFGS) unconstrained optimization method is used to learn optimal weight parameter *ζ* of MEMM. The training process is explained in Algorithm 1.

**Algorithm 1:** MEMM learning (Ψ, Φ).

**begin**

 Initialize *S* ← Ψ = {*χ*_1_, *χ*_2_, …, *χ*_*M*_}

 Randomly select a state *χ*_*i*_

 **while** *S* **do**

  Find all pairs of state-observation (*χ*_*i*_, *φ*_*i*_)

  Consider the selected *χ*_*i*_ as the state Ψ_*i*−1_ in the determining

   *P*(Ψ_*i*_|Ψ_*i*−1_, Φ_*i*_)

  Determine optimal weight parameter *ζ* from Eq (8) through L-BFGS optimization method to maximize the *log* likelihood probability

   *P*(Ψ_*i*_|Ψ_*i*−1_, Φ_*i*_)

  *S* ← *S*\*χ*_*i*_

  Select a state *χ*_*i*_ from *S*

 **end**

**end**

#### Generation of Expression State Sequence through Viterbi Algorithm

Commonly, the Viterbi algorithm is applied in dynamic programming approach (such as finite state Markov process) in order to determine the most likely state sequence by analyzing the corresponding observation sequence. In this work, an improved Viterbi algorithm (as shown in Algorithm 2) is implemented to determine the most likely hidden expression state sequence from a sequence of observations Φ. As described before, extracted features from video frame at time *τ*_*i*_ is considered as observation *φ*_*i*_.

The legacy Viterbi determines most likely hidden expression state sequence through initial, emission and transition probabilities i.e., *P*(*χ*_*i*_), *P*(*φ*_*τ*_|*χ*_*i*_), and *P*(*χ*_*i*_|*χ*_*k*_) respectively. On the other hand, the modified Viterbi employs only a single function *P*(*χ*_*i*_|*χ*_*k*_), *φ*_*τ*_. Hence, Eq (9) is is used to determine the Viterbi value *η*.

$$\eta_{\tau }\left(i\right)=\underset{1\leq k<M}{max}\left(\eta_{\tau -1}\left(k\right)*P\left(\chi_{i}|\chi_{k},\varphi_{\tau }\right)\right)$$ (9)

Here, state *i* lies in 1 ≤ *k* < *M*. However, *P* (*χ*_*i*_|*χ*_*k*_, *φ*_*τ*_) is determined through Eq (3) using optimal parameter *ζ* from the trained system. In respect to observation Φ, the modified Viterbi returns a sequence of most likely expression states *L* = {*l*_1_, *l*_2_, …, *l*_*p*_} ∈ Ψ. Finally, the predicted expression is inferred from the generated of most likely expression state sequence *L* of the overall expression state of ℑ duration.

**Algorithm 2:** Modified Viterbi (Ω, Ψ, Φ).

**begin**

 *M* = |Ψ|

 *i* = 1

 **while** (*i* ≤ *M*) **do**

  *η*_1_(*i*) = *P*(*χ*_*i*_|*φ*_1_)

  *λ*_1_(*i*) = 0

  *i* = *i* + 1

 **end**

 *τ* = 2

 **while**(*τ* ≤ ℑ) **do**

  *i* = 1

  **while**(*i* ≤ *Z*) **do**

   $\eta_{\tau }(i)=\underset{1\leq k<M}{max}(\eta_{\tau -1}(k)*P(\chi_{i}|\chi_{k},\varphi_{\tau }))$

   $\lambda_{\tau }(i)=\underset{1\leq k<M}{argmax}(\eta_{\tau -1}(k)*P(\chi_{i}|\chi_{k},\varphi_{\tau }))$

   *i* = *i* + 1

  **end**

  *τ* = *τ* + 1

 **end**

 $J^{*}=\max_{1\leq k<M}(\eta_{ℑ}(i))$

 $l_{ℑ}=i_{ℑ}^{*}=\underset{1\leq k<M}{\arg\max}(\eta_{ℑ}(i))$

 *τ* = ℑ − 1

 **while** *τ* ≥ 1 **do**

  $i_{\tau }^{*}=\underset{\tau +1}{\lambda }(i_{\tau +1}^{*})$

  $l_{\tau }=i_{\tau }^{*}$

  *τ* = *τ* − 1

 **end**

 return *L*

**end**

#### Prediction of the Expression State

The expression may vary in several video frames of ℑ duration. However, to define expression state of ℑ duration, the cardinality of each state within ℑ is determined. Different states cardinality i,e., |*χ*_1_, *χ*_1_, …, *χ*_*M*_| is measured from *L* and the expression state with highest cardinality is defined as the predicted expression. Algorithm 3 shows stepwise procedure to predict expressions from generated expression states sequence.

**Algorithm 3:** Expression state prediction (Ω, Ψ, Φ, *γ*).

**begin**

 *L* = Viterbi (Ω, Ψ, Φ)

 *M* = |Ψ|

 *i* = 1

 **while** (*i* ≤ *M*) **do**

  *F*_*χ*_*i*__ = 0

  *P* = |*L*|

  *j* = 1

  **while** (*j* ≤ *P*) **do**

   **if** *χ*_*i*_ = = *l*_*j*_ **then**

    *F*_*χ*_*i*__ = *F*_*χ*_*i*__ + 1

   **end**

  **end**

  |*χ*_*i*_| = *F*_*χ*_*i*__

 **end**

 $\hat{\chi }=arg\underset{\chi_{i}}{max}|\chi_{i}|$

 *i* = 1

 **while** (*i* ≤ *M*) **do**

  **if** |*χ*_*i*_| > *γ*_1_ && $\hat{\chi }\in${*’Happy’*}**then**

   return *χ*_*i*_

  **end**

  **else if** |*χ*_*i*_| > *γ*_2_ && $\hat{\chi }\in${*’Anger’*}**then**

   return $\hat{\chi }$

  **end**

  **else if** |*χ*_*i*_| > *γ*_3_ && $\hat{\chi }\in${*’Sad’*}**then**

   return $\hat{\chi }$

  **end**

  **else if** |*χ*_*i*_| > *γ*_4_ && $\hat{\chi }\in${*’Surprise’*}**then**

   return $\hat{\chi }$

  **end**

  **else if** |*χ*_*i*_| > *γ*_5_ && $\hat{\chi }\in${*’Fear’*}**then**

   return $\hat{\chi }$

  **end**

  **else if** |*χ*_*i*_| > *γ*_6_ && $\hat{\chi }\in${*’Disgust’*}**then**

   return $\hat{\chi }$

  **end**

  **else**

   return $\arg\max_{\chi_{i}}|\chi_{i}|$

  **end**

 **end**

**end**

## System Validation

### Datasets Used

For performance evaluation, six publicly available standard datasets of facial expressions are used, which are as follows.

- *Extended Cohn-Kanade Dataset (CK+)*:
  This dataset contains 593 videos sequences comprising seven facial expressions recorded by 123 subjects (university students) [34]. The subjects include majority of female students with age range from 18 to 30 years. Out of total 593 sequences, 309 sequences are used in this work. Out of seve, six expressions are used for evaluation. The size of each frame is 640×480 pixels in some images, and 640×490 pixels in others with 8-bits precision for gray-scale values. This dataset is publicly available and can be found using http://www.consortium.ri.cmu.edu/ckagree/. This dataset belongs to Carnegie Mellon University, USA.
- *Japanese Female Facial Expression (JAFFE) Dataset*:
  The expressions in this dataset were collected from 10 different (Japanese female) subjects [35]. Each image has been rated on six expression adjectives by 60 Japanese subjects. Most of the expression frames were taken from the front view of the camera with tied hair in order to expose the entire face. This dataset consists of 213 facial frames and has seven expressions, including the neutral expression. Out of these, 193 facial frames for six facial expressions are used. The size of each facial frame is 256×256 pixels. This dataset can be downloaded by using http://www.kasrl.org/jaffe.html. This dataset belongs to Ritsumeikan University, Kyoto, Japan.
- *Multimedia Understanding Group (MUG) Dataset*:
  In this dataset, 86 subjects performed six expressions with constant blue background with the frontal view of the camera [36]. Two light sources of 300W each, mounted on stands at a height of 130cm approximately were used. A predefined setup with the help of umbrella was utilized in order to diffuse light and avoid shadow. The images were captured at a rate of 19 frames per second. The original size of each image is 896×896 pixels. The dataset is available in http://mug.ee.auth.gr/fed/. This dataset belongs to Aristotle University of Thessaloniki, Thessaloniki, Greece.
- *USTC-NVIE spontaneous-based Dataset*:
  In USTC-NVIE dataset, an infrared thermal and a visible camera was used in order to collect both spontaneous and posed expressions, but in this work, we only utilize the spontaneous-based expressions [37]. There were a total 105 subjects. They performed a series of expressions with illumination from three different directions: front illumination, left illumination, and right illumination. Subjects’ age range was from 17 to 31 years. Some of them worn glasses, whereas others were without glasses. The size of each facial frame is 640×480 or 704×490 pixels. In total, 910 expression frames are utilized from this dataset. This facial expression dataset is publicly available in http://nvie.ustc.edu.cn/index.html. This dataset belongs to University of Science and Technology, Hefei, Anhui, P.R. China.
- *Indian Movie Face Database (IMFDB)*:
  The IMFDB dataset was collected from Indian movies of different languages [38]. Most of the videos were collected from the last two decades which contain large diversity in illumination, and image resolution. In IMFDB, the subjects wore partial or full-makeup. The images are from frontal, left, right, up, and down views of camera. The dataset has basic six expressions captured from 67 male and 33 female actors of different age groups, such as children (1–12 years), young adults (13–30 years), middle aged (31–50 years), and elderly (Above 50 years) with at least 200 images from each actor. Some subjects wore glasses and had beard, ornaments, hair, hand, or none. In order to maintain consistency among the images, a heuristic method for cropping is applied, and all the images are manually selected and cropped from the video frames. The size of each image which we used for our experiments is 140×180 pixels. The dataset can be downloaded by using http://cvit.iiit.ac.in/projects/IMFDB/, which belongs to Indian Institute of Information Technology, Hyderabad, India.
- Acted Facial Expressions in the Wild Database (AFEW):
  AFEW dataset [39] is publicly available standard dataset that has been collected from movies in indoor and outdoor (real world) environments. The age range of the subjects were from 1-70 years. All the expression related information such as name, age, pose, gender, expression type, etc were stored in XML schema. Static Facial Expressions in the Wild (SFEW) has been developed by selecting frames from AFEW. The database covers unconstrained facial expressions, varied head poses, large age range, occlusions, varied focus, different resolution of face and close to real world illumination. Frames were extracted from AFEW sequences and labelled based on the label of the sequence. In total, SFEW contains 700 images and which include seven basic expressions happy, anger, sad, surprise, fear, disgust, and neutral. But, we have selected the six basic expressions excluding neutral for fair comparison. The AFEW dataset of facial expression can be downloaded by using https://cs.anu.edu.au/few/AFEW.html, and the dataset belongs to University of Miami, Florida, USA.

It should be noted that since each dataset contains different expressions, six common expressions among them are selected for this work. These are happy, anger, sad, surprise, fear, and disgust. Furthermore, the datasets contain a high degree of variability in terms of scale, pose, illumination, resolution, occlusion, makeup, age and other physical characteristics of the participants. It is this high degree of variance which usually results in degrading the performance of and FER system when tested for different datasets.

### Experimental Setup

For a thorough validation, the following set of four experiments is performed, and all the experiments are performed in Matlab using an Intel Core^™^ i7-6700 (3.4 GHz) with a RAM capacity of 16 GB.

- In the first experiment, performance of the proposed model is analyzed on each dataset using a 10–fold cross-validation scheme. In other words, each dataset is divided into ten equal parts. From these, one is used for testing; whereas, the remaining nine are used for training the system.
- In the second experiment, the robustness of the proposed model is assessed. For this experiment, out of six datasets, one dataset is used for training; whereas, the other five datasets are used for testing purpose. This process is repeated six times so that each dataset is used exactly once as the training dataset.
- In the third experiment, the setup of the first experiment is repeated; however, the classification module, i.e., MEMM is replaced with HMM. The purpose is to evaluate the performance of the proposed classification model against the traditionally used model, i.e., HMM.
- Finally, in the fourth experiment, the proposed FER system is compared against state-of-the-art systems for FER.

## Results and Discussion

### First Experiment

**Fig 4:**
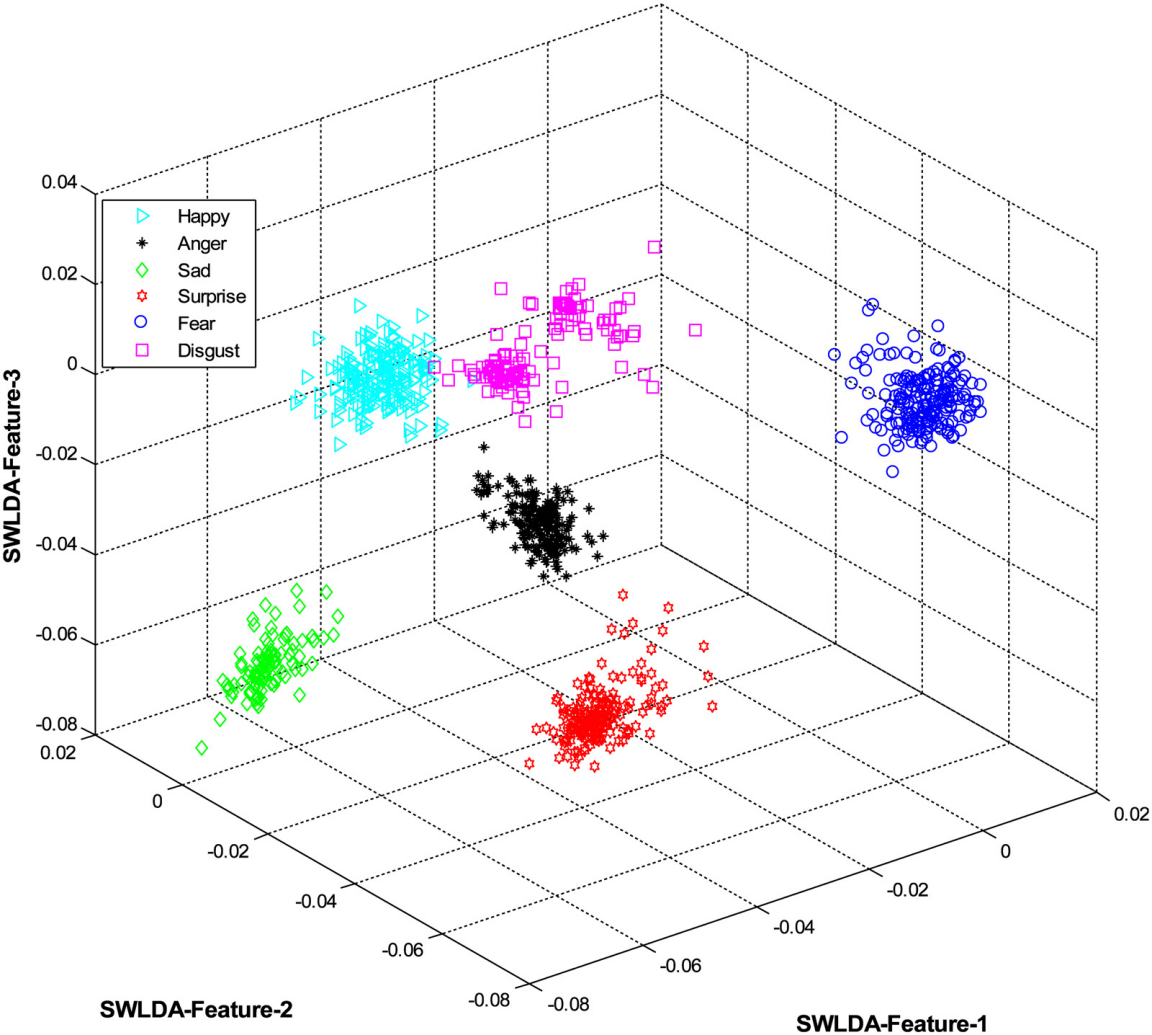
3D-feature plot of the proposed FER system for the six facial expressions using CK+ dataset. It can be seen that the system clearly classified the expressions classes.

**Fig 5:**
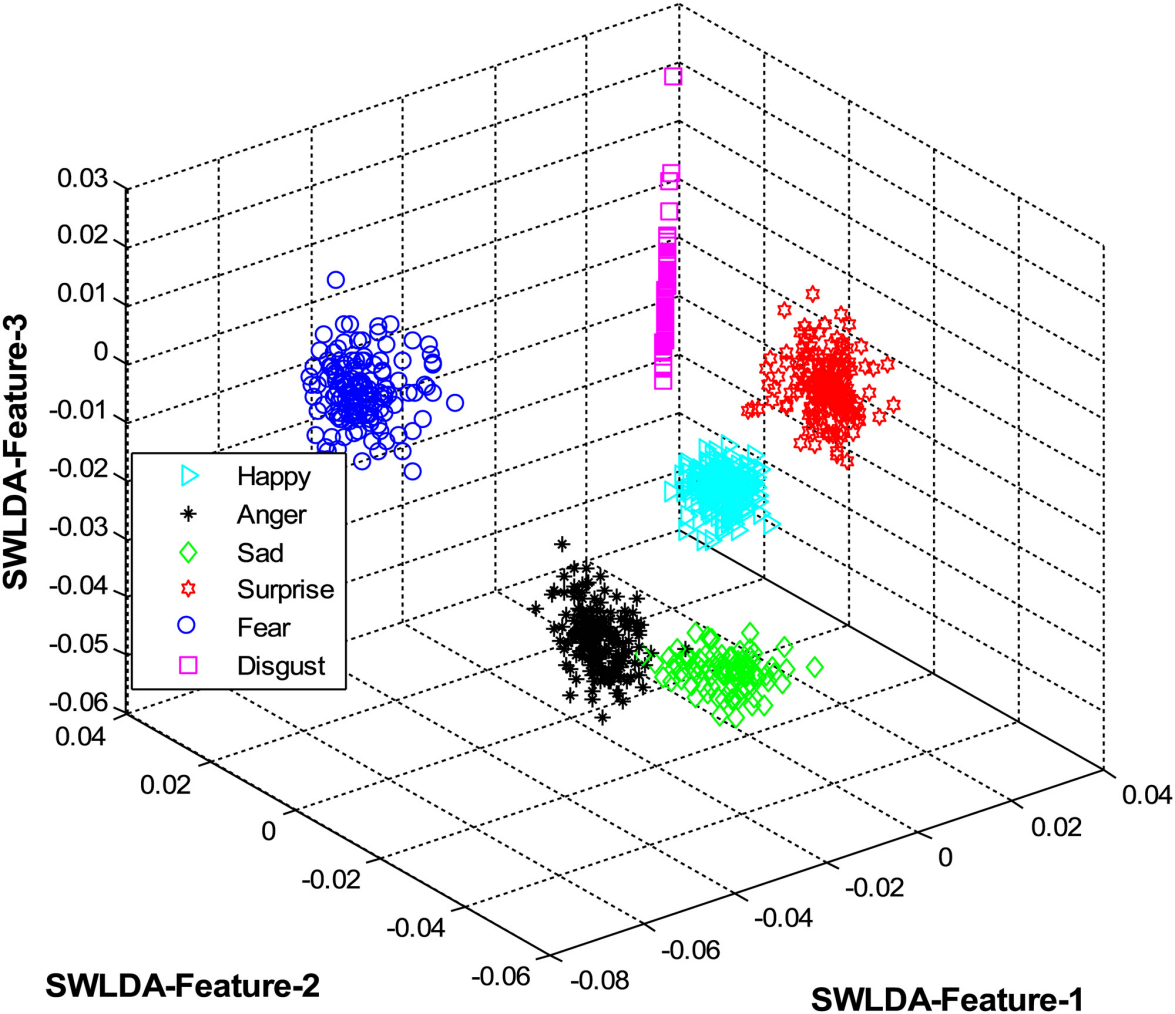
3D-feature plot of the proposed FER system for the six facial expressions using JAFFE dataset. It can be seen that the system clearly classified the expressions classes.

**Fig 6:**
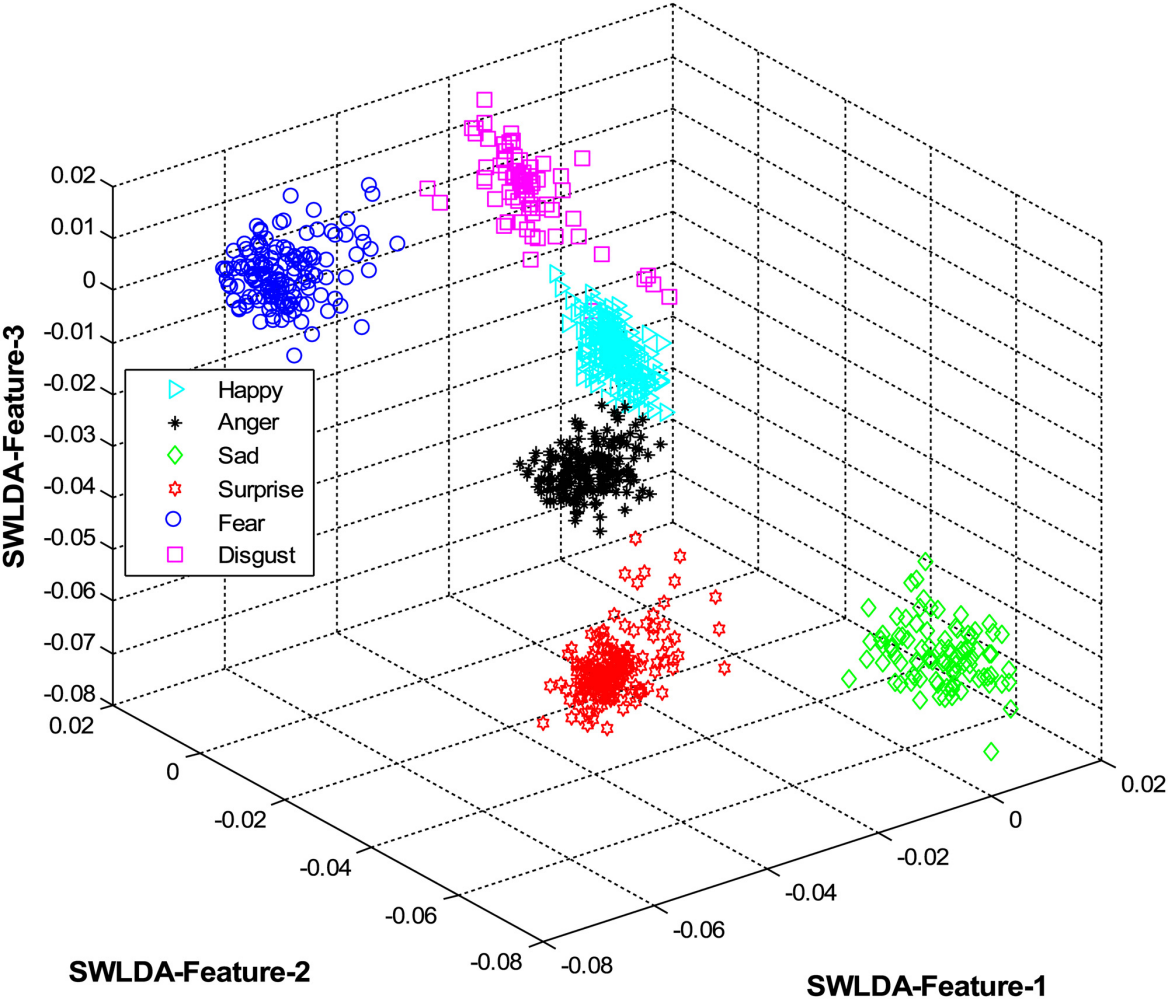
3D-feature plot of the proposed FER system for the six facial expressions using MUG dataset. It can be seen that the system clearly classified the expressions classes.

**Fig 7:**
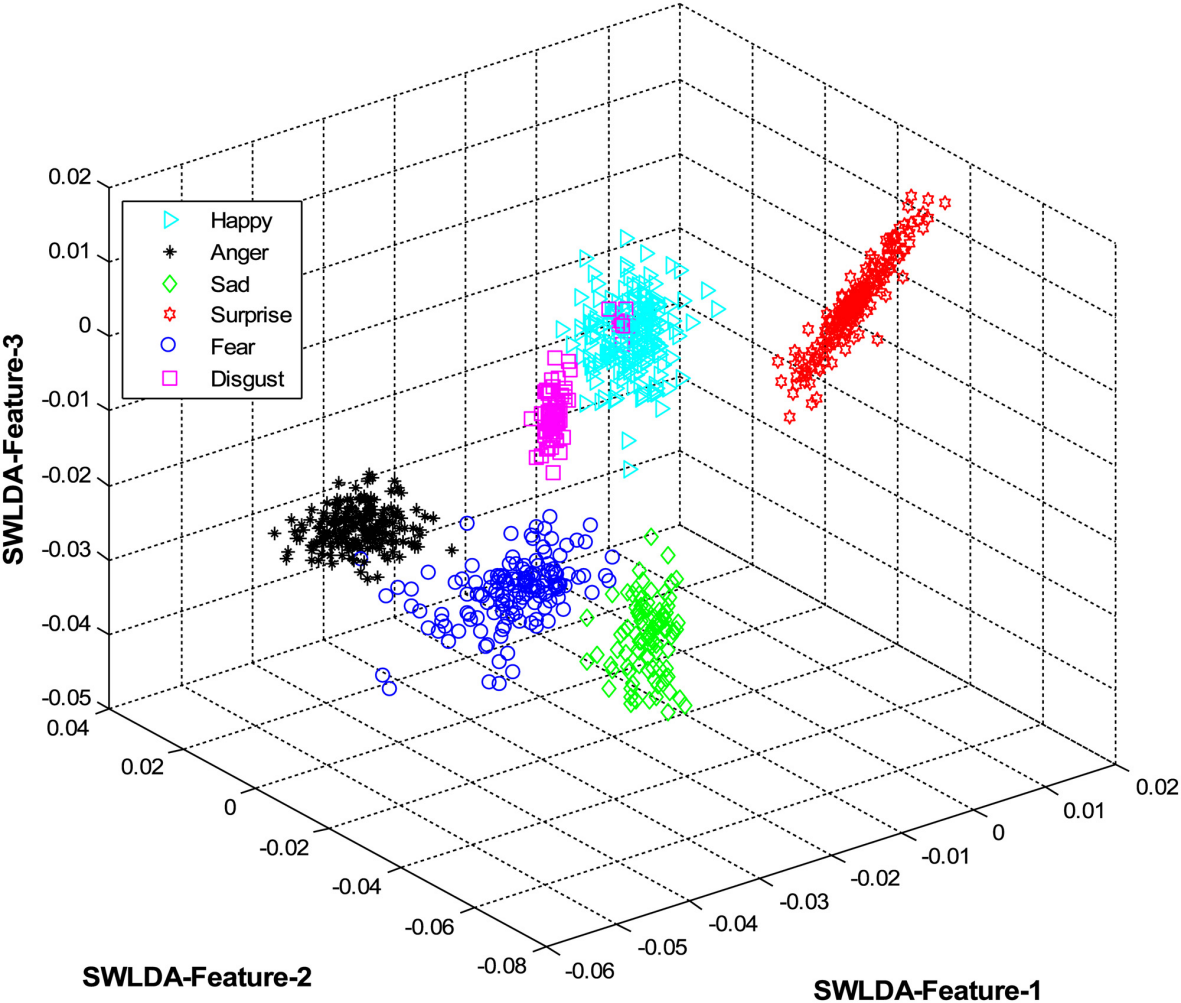
3D-feature plot of the proposed FER system for the six facial expressions using USTC-NVIE dataset. It can be seen that the system clearly classified the expressions classes.

**Fig 8:**
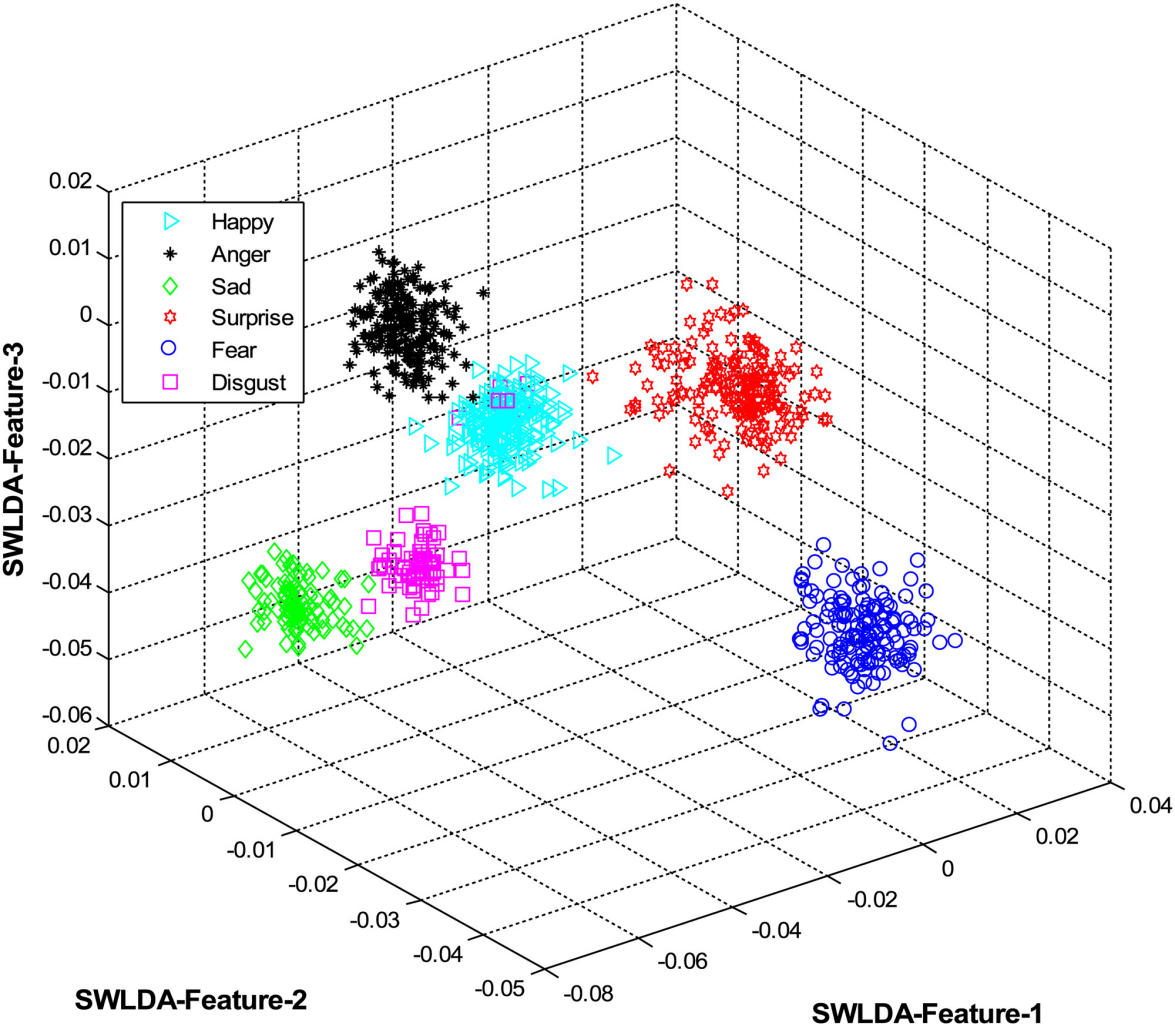
3D-feature plot of the proposed FER system for the six facial expressions using IMFDB dataset. It can be seen that the system clearly classified the expressions classes.

**Fig 9:**
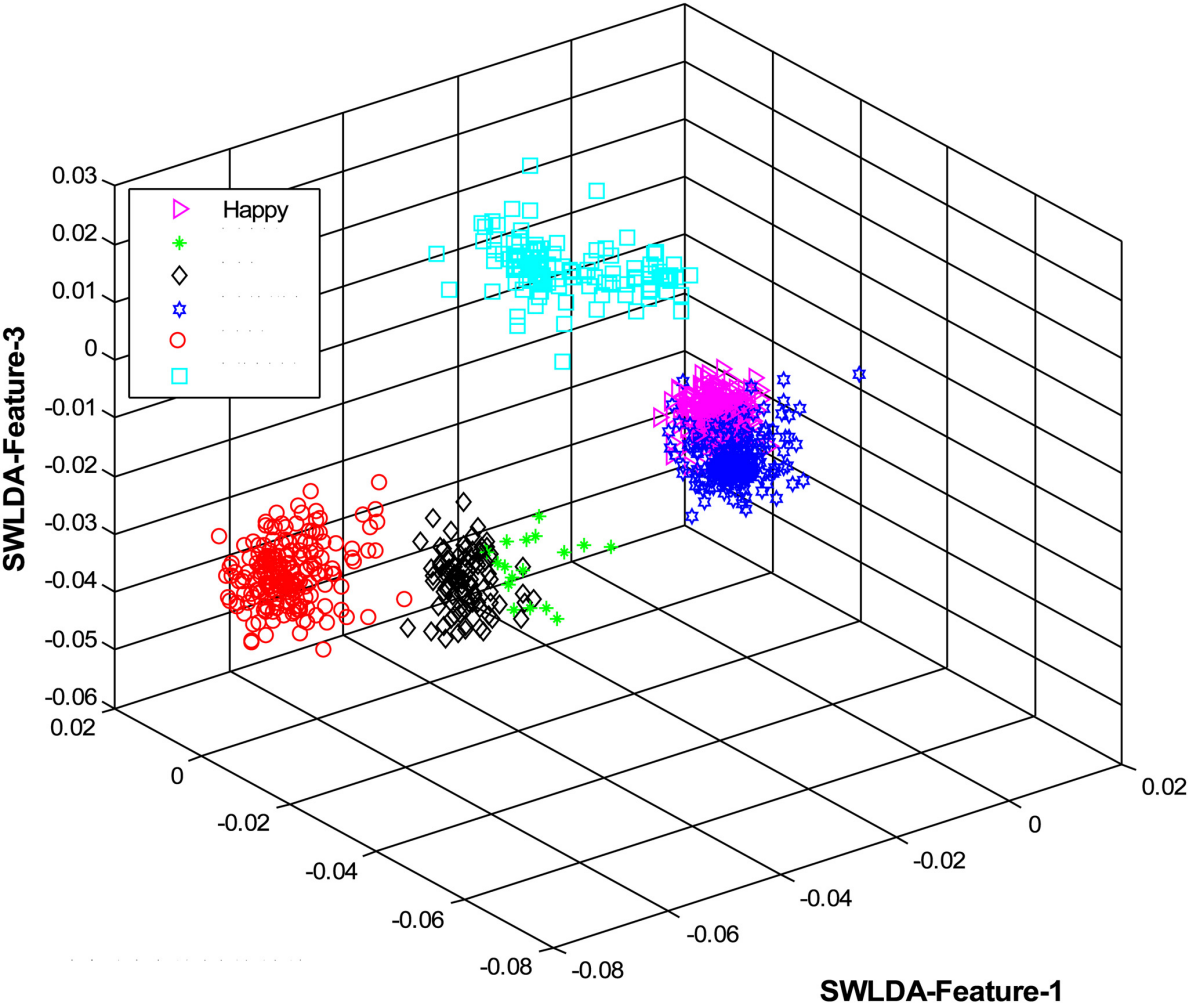
3D-feature plot of the proposed FER system for the six facial expressions using AFEW dataset. It can be seen that the system clearly classified the expressions classes.

The overall results are shown in Table 1 and Fig 4 (using CK+ dataset), Table 2 and Fig 5 (using JAFFE dataset), Table 3 and Fig 6 (using MUG dataset), Table 4 and Fig 7 (using USTC-NVIE dataset), Table 5 and Fig 8 (using IMFDB dataset) and Table 6 and Fig 9 respectively.

**Table 1:** Recognition rate of the proposed FER system using CK+ dataset of facial expressions (Unit: %).

**Expressions**	Happy	Anger	Sad	Surprise	Fear	Disgust
Happy	**97**	1	2	0	0	0
Anger	0	**98**	1	0	1	0
Sad	0	2	**97**	0	1	0
Surprise	0	2	0	**98**	0	0
Fear	0	0	0	0	**100**	0
Disgust	0	0	1	0	0	**99**
Average	**98.16**					

**Table 2:** Recognition rate of the proposed FER system using JAFFE dataset of facial expressions (Unit: %).

**Expressions**	Happy	Anger	Sad	Surprise	Fear	Disgust
Happy	**100**	0	0	0	0	0
Anger	0	**98**	0	1	0	1
Sad	1	0	**97**	2	0	0
Surprise	0	1	0	**99**	0	0
Fear	0	1	0	2	**96**	1
Disgust	0	0	0	0	0	**100**
Average	**98.33**					

**Table 3:** Recognition rate of the proposed FER system using MUG dataset of facial expressions (Unit: %).

**Expressions**	Happy	Anger	Sad	Surprise	Fear	Disgust
Happy	**96**	1	1	1	1	0
Anger	0	**98**	0	1	0	1
Sad	0	0	**99**	1	0	0
Surprise	1	1	2	**96**	0	0
Fear	0	3	0	0	**97**	0
Disgust	1	0	0	2	0	**97**
Average	**97.20**					

**Table 4:** Recognition rate of the proposed FER system using USTC-NVIE dataset of facial expressions (Unit: %).

**Expressions**	Happy	Anger	Sad	Surprise	Fear	Disgust
Happy	**100**	0	0	0	0	0
Anger	0	**97**	1	0	2	0
Sad	0	1	**97**	1	0	1
Surprise	0	1	0	**99**	0	0
Fear	0	2	0	0	**98**	0
Disgust	0	0	0	0	0	**100**
Average	**98.50**					

**Table 5:** Recognition rate of the proposed FER system using IMFDB dataset of facial expressions (Unit: %).

**Expressions**	Happy	Anger	Sad	Surprise	Fear	Disgust
Happy	**95**	1	2	1	0	1
Anger	0	**97**	0	0	3	0
Sad	0	1	**96**	2	1	0
Surprise	1	2	1	**94**	2	0
Fear	0	1	0	0	**99**	0
Disgust	0	1	1	1	0	**97**
Average	**96.33**					

**Table 6:** Recognition rate of the proposed FER system using AFEW dataset of facial expressions (Unit: %).

**Expressions**	Happy	Anger	Sad	Surprise	Fear	Disgust
Happy	**93**	3	1	0	2	1
Anger	1	**96**	1	1	0	1
Sad	1	2	**91**	1	2	3
Surprise	0	1	0	**98**	0	1
Fear	0	0	2	1	**96**	1
Disgust	1	0	2	0	2	**95**
Average	**94.83**					

It is obvious from Tables 1, 2, 3, 4, 5 and 6 that the proposed model constantly displayed a high recognition accuracy on all datasets. That is, 98.16% on CK+ dataset, 98.33% on JAFFE dataset, 97.20% on MUG dataset, 98.50% on USTC-NVIE dataset, 96.33% on IMFDB dataset, and 94.83% on AFEW dataset.

### Second Experiment

The overall results for this experiment are represented in Tables 7, 8, 9, 10, 11 and 12. It can be seen from Tables 7, 9 and 10 that a better performance is achieved when the system is trained using CK+, MUG, and USTC-NVIE datasets. On the other hand, the accuracy decreased slightly when the system is trained on JAFFE and IMFDB datasets (as shown in Tables 8, 11 and 12). The reason for this is different eye features, camera orientation, and wearing of glasses. In JAFFE dataset, eye features of subjects are significantly different from the subjects in datasets. The expressions in IMFDB and AFEW datasets are captured from various angles as opposed to the other datasets, where a front-view is mostly used. Also, some subjects in IMFDB and AFEW have glasses in dynamic scenarios, too. Nevertheless, the results are very encouraging and suggest that the proposed FER system is robust. That is, the proposed FER system showed better performance not only on one dataset but also across multiple datasets, which is one the major limitations of existing works.

**Table 7:** Confusion matrix of the proposed FER system that is trained on CK+ dataset and tested on JAFFE, MUG, USTC-NVIE, IMFDB, and AFEW datasets of facial expressions (Unit: %).

**Expressions**	Happy	Anger	Sad	Surprise	Fear	Disgust
Happy	**83**	3	4	4	4	2
Anger	3	**84**	6	3	4	0
Sad	1	3	**88**	4	1	3
Surprise	2	3	2	**89**	2	2
Fear	2	3	2	2	**90**	1
Disgust	3	3	4	3	3	**84**
Average	**86.33**					

**Table 8:** Confusion matrix of the proposed FER system that is trained on JAFFE dataset and tested on CK+, MUG, USTC-NVIE, IMFDB, and AFEW datasets of facial expressions (Unit: %).

**Expressions**	Happy	Anger	Sad	Surprise	Fear	Disgust
Happy	**82**	4	5	2	4	3
Anger	2	**85**	3	2	5	3
Sad	5	5	**81**	3	4	3
Surprise	3	2	2	**87**	2	4
Fear	5	2	3	2	**86**	2
Disgust	4	2	3	5	3	**83**
Average	**84.00**					

**Table 9:** Confusion matrix of the proposed FER system that is trained on MUG dataset and tested on CK+, JAFFE, USTC-NVIE, IMFDB, and AFEW datasets of facial expressions (Unit: %).

**Expressions**	Happy	Anger	Sad	Surprise	Fear	Disgust
Happy	**89**	2	3	1	2	3
Anger	3	**87**	2	4	1	3
Sad	4	3	**84**	3	4	2
Surprise	3	4	3	**83**	2	5
Fear	1	2	3	3	**88**	3
Disgust	2	2	4	3	3	**86**
Average	**86.17**					

**Table 10:** Confusion matrix of the proposed FER system that is trained on USTC-NVIE dataset and tested on CK+, JAFFE, MUG, IMFDB, and AFEW datasets of facial expressions (Unit: %).

**Expressions**	Happy	Anger	Sad	Surprise	Fear	Disgust
Happy	**91**	1	3	2	0	3
Anger	4	**85**	2	2	4	3
Sad	3	2	**88**	3	3	1
Surprise	1	2	3	**90**	2	2
Fear	3	2	2	3	**86**	4
Disgust	1	1	2	3	5	**88**
Average	**88.00**					

**Table 11:** Confusion matrix of the proposed FER system that is trained on IMFDB dataset and tested on CK+, JAFFE, MUG, USTC-NVIE, and AFEW datasets of facial expressions (Unit: %).

**Expressions**	Happy	Anger	Sad	Surprise	Fear	Disgust
Happy	**85**	5	3	2	4	1
Anger	3	**86**	2	3	2	4
Sad	5	2	**87**	3	2	1
Surprise	1	3	3	**81**	2	4
Fear	1	3	2	4	**86**	4
Disgust	3	3	4	3	5	**82**
Average	**84.50**					

**Table 12:** Confusion matrix of the proposed FER system that is trained on AFEW dataset and tested on CK+, JAFFE, MUG, USTC-NVIE, and IMFDB datasets of facial expressions (Unit: %).

**Expressions**	Happy	Anger	Sad	Surprise	Fear	Disgust
Happy	**80**	3	5	3	4	5
Anger	3	**85**	4	2	4	2
Sad	3	3	**84**	4	2	4
Surprise	3	2	4	**83**	3	5
Fear	3	4	3	2	**82**	6
Disgust	4	1	4	2	3	**86**
Average	**83.33**					

### Third Experiment

The overall set of results are shown in Tables 13, 14, 15, 16, 17 and 18. It can be seen that the MEMM model played a significant role in achieving the higher recognition rates in the first experiment. When it is replaced with HMM, the system is unable to display the same high performance under the exact same settings. Thus this experiment validates our hypothesis and provides clear evidence that MEMM based recognition model has the capability to accurately classify expressions in both spontaneous and natural environments.

**Table 13:** Confusion matrix of the proposed FER system with HMM (as a recognition model), instead of using the proposed recognition model (that is MEMM model) using CK+ dataset of facial expressions (Unit: %).

**Expressions**	Happy	Anger	Sad	Surprise	Fear	Disgust
Happy	**93**	0	2	3	0	2
Anger	0	**95**	1	2	1	1
Sad	2	2	**92**	1	2	1
Surprise	2	2	0	**94**	2	0
Fear	1	2	2	0	**92**	3
Disgust	2	2	1	2	3	**90**
Average	**92.66**					

**Table 14:** Confusion matrix of the proposed FER system with HMM (as a recognition model), instead of using the proposed recognition model (that is MEMM model) using JAFFE dataset of facial expressions (Unit: %).

**Expressions**	Happy	Anger	Sad	Surprise	Fear	Disgust
Happy	**92**	1	2	1	3	1
Anger	0	**95**	3	2	0	0
Sad	1	3	**96**	0	0	0
Surprise	2	3	2	**91**	1	1
Fear	1	4	1	2	**90**	2
Disgust	0	0	0	0	5	**95**
Average	**93.16**					

**Table 15:** Confusion matrix of the proposed FER system with HMM (as a recognition model), instead of using the proposed recognition model (that is MEMM model) using MUG dataset of facial expressions (Unit: %).

**Expressions**	Happy	Anger	Sad	Surprise	Fear	Disgust
Happy	**94**	2	1	1	2	0
Anger	3	**89**	1	2	2	3
Sad	1	2	**92**	0	2	3
Surprise	2	0	1	**93**	4	0
Fear	0	0	1	2	**95**	2
Disgust	0	1	1	1	3	**94**
Average	**92.83**					

**Table 16:** Confusion matrix of the proposed FER system with HMM (as a recognition model), instead of using the proposed recognition model (that is MEMM model) using USTC-NVIE dataset of facial expressions (Unit: %).

**Expressions**	Happy	Anger	Sad	Surprise	Fear	Disgust
Happy	**92**	2	1	2	1	2
Anger	1	**96**	1	1	1	0
Sad	0	0	**95**	1	2	2
Surprise	2	3	1	**88**	2	4
Fear	0	0	0	3	**94**	3
Disgust	1	1	2	1	2	**93**
Average	**93.00**					

**Table 17:** Confusion matrix of the proposed FER system with HMM (as a recognition model), instead of using the proposed recognition model (that is MEMM model) using IMFDB dataset of facial expressions (Unit: %).

**Expressions**	Happy	Anger	Sad	Surprise	Fear	Disgust
Happy	**90**	2	3	1	2	2
Anger	0	**92**	4	2	2	0
Sad	2	2	**91**	3	2	0
Surprise	2	3	1	**89**	2	3
Fear	0	0	0	2	**94**	4
Disgust	0	1	1	1	3	**95**
Average	**91.83**					

**Table 18:** Confusion matrix of the proposed FER system with HMM (as a recognition model), instead of using the proposed recognition model (that is MEMM model) using AFEW dataset of facial expressions (Unit: %).

**Expressions**	Happy	Anger	Sad	Surprise	Fear	Disgust
Happy	**89**	1	2	3	1	4
Anger	2	**87**	3	3	4	1
Sad	4	3	**88**	2	1	2
Surprise	0	3	3	**90**	2	2
Fear	1	2	3	1	**91**	2
Disgust	1	2	3	0	2	**92**
Average	**89.50**					

### Fourth Experiment

As stated earlier, in this experiment, the proposed FER system (including the MEMM model) is compared with some stat-of-the-art works: [16, 18, 40–42]. For this experiment, all the datasets are utilized under. For some works, the code is obtained and actual results are reported; whereas, for the others, the published results are reported. For each dataset, the same 10-fold cross-validation scheme is used as in the first experiment. The weighted average recognition rate of the existing works and that of the proposed FER system on all the datasets are shown in Table 19. It can be seen that the proposed FER system, with the MEMM model, achieved higher recognition rate than all the existing stat-of-the-art works on all the datasets. This proves its ability to accurately and robustly recognize facial expressions from video data.

**Table 19:** Comparison results of the proposed FER system with the proposed MEMM model against some stat-of-the-art works (Unit: %).

Previous FER Systems	Average Recognition Rates	Standard Deviation
[40]	82	±3.1
[41]	88	±4.5
[16]	87	±3.7
[42]	92	±2.1
[18]	90	±1.9
Proposed FER System	**97**	±1.3

## Conclusion and Future Directions

Expressions play a significant role in determining the attitude and behavior of a human. FER systems have been proposed previously; however, accurate and robust FER is still a major challenge for such systems. In most case, the recognition accuracy of existing works degrade in spontaneous environments. Furthermore, variance due to illumination changes, pose, camera angle, etc., limits their use in different scenarios. Accordingly, in this paper, a new MEMM base FER system is proposed. In this model, the states of the human expressions are modeled as the states of maximum entropy Markov model (MEMM), in which the video-sensor observations are considered as the observations of MEMM. A modified Viterbi, a machine-learning algorithm, is used to generate the most probable expression state sequence based on such observations; then, from the most likely state sequence, the expression state is predicted through the proposed algorithm. Unlike most of the existing works, which were evaluated using a single dataset, performance of the proposed approach is assessed in a large-scale experimentation using six publicly available spontaneous datasets in order to show the robustness of the proposed model. The proposed approach showed better performance against existing state-of-the-art methods and achieved a weighted average recognition rate of 97% across all the datasets.

In most of the existing datasets, RGB cameras were utilized which may raise privacy concern; therefore, in order to solve this concern, a depth camera will be utilized in the further study. Improvements will be made in the algorithms and methods to ensure the same performance and robustness in the case of depth-images, too.
